# Performance of ChatGPT and DeepSeek in the Management of Postprostatectomy Urinary Incontinence

**DOI:** 10.1590/S1677-5538.IBJU.2025.0325

**Published:** 2025-08-10

**Authors:** Vicktor Bruno Pereira Pinto, Romullo José Costa Ataídes, Lucas Antônio Pereira do Nascimento, Cristiane de Barros Gaspar, Priscila Ferreira Alves, Magnum Adriel Santos Pereira, Manoel José de Macedo, Willian Carlos Nahas, José de Bessa, Cristiano Mendes Gomes

**Affiliations:** 1 Faculdade de Medicina da Universidade de São Paulo Divisão de Urologia São Paulo Brasil Divisão de Urologia, Faculdade de Medicina da Universidade de São Paulo - FMUSP, São Paulo, Brasil;; 2 Centro Universitário UniDombosco São Luis MA Brasil Centro Universitário UniDombosco - UNDB, São Luis, MA, Brasil;; 3 Serviço de Urologia do Hospital do Servidor Público Estadual de São Paulo São Paulo SP Brasil Serviço de Urologia do Hospital do Servidor Público Estadual de São Paulo -HSPE - IAMSPE, São Paulo, SP, Brasil;; 4 Universidade Estadual de Feira de Santana Departamento de Cirurgia Feira de Santana BA Brasil Departamento de Cirurgia, Universidade Estadual de Feira de Santana - UEFS, Feira de Santana, BA, Brasil

**Keywords:** Artificial Intelligence, Urinary Incontinence, Clinical Decision-Making

## Abstract

**Purpose:**

Artificial intelligence (AI) continues to evolve as a tool in clinical decision support. Large language models (LLMs), such as ChatGPT and DeepSeek, are increasingly used in medicine to provide fast, accessible information. This study aimed to compare the performance of ChatGPT and DeepSeek in generating recommendations for the management of postprostatectomy urinary incontinence (PPUI), based on the AUA/SUFU guideline.

**Materials and Methods:**

A total of 20 questions (10 conceptual and 10 case-based) were developed by three urologists with expertise in PPUI, following the AUA/SUFU guideline. Each question was submitted in English using zero-shot prompting to ChatGPT-4o and DeepSeek R1. Responses were limited to 200 words and graded independently as correct (1 point), partially correct (0.5), or incorrect (0). Total and domain-specific scores were compared.

**Results:**

ChatGPT achieved 19 out of 20 points (95.0%), while DeepSeek scored 14.5 (72.5%; p = 0.031). In conceptual questions, scores were 9.0 (ChatGPT) and 8.0 (DeepSeek; p = 0.50). In case-based scenarios, ChatGPT scored 10.0 versus 6.5 for DeepSeek (p = 0.08). ChatGPT outperformed DeepSeek across all guideline domains. DeepSeek made critical errors in the treatment domain, such as recommending a male sling for radiated patients.

**Conclusion:**

ChatGPT demonstrated superior performance in providing guideline-based recommendations for PPUI. However, both models should be used under expert supervision, and future research is needed to optimize their safe integration into clinical workflows.

## INTRODUCTION

Artificial intelligence (AI) has emerged as a transformative force in healthcare, particularly through large language models (LLMs) capable of processing and generating complex medical content. ChatGPT, developed by OpenAI, has become one of the most widely used tools, designed to simulate human-like dialogue and provide accurate, contextually relevant responses (1). Since its release in 2022, ChatGPT has shown substantial potential in supporting healthcare professionals by enabling rapid access to clinical information, assisting in medical decision-making, and improving patient care workflows (2-5).

More recently, other LLMs such as Perplexity, Gemini 2.0 and Copilot have entered the field, offered unique features and expanded the landscape of AI in medicine (6). In mid-January 2025, DeepSeek-R1, an innovative open-source LLM that has rapidly gained prominence worldwide, is an open-source and cost-free platform with strong performance and broad accessibility (7). Its disruptive potential lies in combining high-quality outputs with zero user cost, making it especially appealing in low-resource settings or for professionals seeking efficient, freely available AI solutions.

As the use of these tools becomes more widespread in clinical and academic settings, their evaluation is essential to ensure safe and practical application in real-world scenarios. For instance, recent studies have evaluated the accuracy and reproducibility of ChatGPT in answering questions related to various urological diagnoses, highlighting areas where its responses can be incomplete or misleading (8). Physicians increasingly turn to LLMs such as ChatGPT, DeepSeek, and Gemini to answer questions across medical specialties, including disease management strategies (4-6, 8). These platforms offer structured, on-demand access to medical knowledge, assisting with treatment algorithms, diagnostic decisions, and drug information retrieval during clinical routines (9, 10)

Urinary incontinence after prostate surgery – particularly postprostatectomy urinary incontinence (PPUI) – remains a common and challenging condition (11). Its management often requires individualized approaches based on various patient factors, and may prompt complex clinical questions. To support clinicians, the Incontinence After Prostate Treatment guidelines developed by the American Urological Association (AUA) and the Society of Urodynamics, Female Pelvic Medicine and Urogenital Reconstruction (SUFU) provide structured, evidence-based recommendations (12)

Our group previously assessed the performance of ChatGPT versions 3.5 and 4 in delivering guideline-based recommendations for PPUI management, using the AUA/SUFU document as a reference standard (5). Building on this foundation, we postulated that ChatGPT-4.0, given its advanced development and optimization, would outperform the recently introduced open-source model DeepSeek R1 in delivering recommendations consistent with established clinical guidelines. Consequently, the present study compares the most recent version of ChatGPT (4o) with DeepSeek (R1), focusing on their ability to deliver accurate and clinically relevant guidance for evaluating and treating PPUI.

## MATERIALS AND METHODS

This research presents a comparative assessment of two widely utilized large language models – ChatGPT-4.0 and DeepSeek R1 – focusing on their capacity to deliver recommendations aligned with established guidelines for managing PPUI. To accomplish this, a set of questions was constructed by three urologists, each possessing over two decades of clinical experience and specialized expertise in PPUI. The questions were derived from the Incontinence After Prostate Treatment: AUA/SUFU Guideline, ensuring that each inquiry corresponded to a well-defined and non-controversial answer (12). The guideline itself is structured into several key domains: (a) considerations before prostate treatment, (b) management following prostate treatment, (c) evaluation strategies for post-treatment incontinence, (d) therapeutic interventions, (e) surgical complications, and (f) complex scenarios, including compromised urethral integrity, bladder neck strictures, and approaches to complications arising from surgical management of PPUI (12)

**Test 1 – Conceptual questions:** Ten conceptual questions were prepared based on the Guideline recommendations, divided into pre-prostate treatment (two questions), post-prostate treatment (two questions), evaluation of incontinence after prostate treatment (three questions), and treatment options (three questions).

**Test 2 – Case-based questions:** To analyze the ability of ChatGPT and DeepSeek to apply knowledge and critical thinking skills, 10 questions were created using real or hypothetical clinical cases and grounded in the concepts and recommendations provided by the AUA guideline. These questions were divided into post-prostate treatment (one question), evaluation of incontinence after prostate treatment (two questions), treatment options (four questions), surgical complications (two questions), and special situations (one question). A list of the questions used in tests 1 and 2 can be found in the *Supporting Information.*

All questions were open-ended and descriptive. They were entered individually and anonymously (without IP tracking) into ChatGPT-4o and DeepSeek R1 in February 2025. A single investigator submitted all prompts in English, who instructed the AI engines to provide specific, concise answers limited to 200 words. The AI models were not prompted to incorporate any particular guidelines. Each question was submitted independently using the "New Chat" function to ensure a zero-shot format, meaning no prior context or sequential prompting was used.

The same three expert urologists who formulated the questions independently evaluated the AI-generated responses. Each answer was graded using the following system:

- (A) Correct (1 point)
- (B) Mixed: includes correct and incorrect or outdated information (0.5 points)
- (C) Incorrect (0 points)

The overall performance of each model—ChatGPT and DeepSeek—was assessed for both conceptual (Test 1) and case-based (Test 2) questions, with a maximum score of 10 points per test.

This study was exempt from IRB review as no patient-level data were used.

### Statistical analysis

Quantitative variables were expressed as absolute values, percentages, or proportions. We compared categorical variables using the Chi‐square test or Fisher's exact test. All tests were two‐sided, and statistical significance was p < 0.05. The analysis was performed using GraphPad Prism, version 10.0.01 for Windows.

## RESULTS

ChatGPT scored 19 out of 20 points (95.0% accuracy). In comparison, DeepSeek scored 14.5 out of 20 points (72.5%; p = 0.031). Tables 1 and 2 show examples of errors and differences in performance between the two AI models. They also show the domains and reasons for all incorrect or partially correct responses from ChatGPT and DeepSeek.

**Table 1:** Sample Questions Showing Divergent Performance Between ChatGPT-4 and DeepSeek in PPUI Recommendations.

Question	Guideline recommendation*	ChatGPT answer	DeepSeek answer
Conceptual question*			
What are the known preoperative risk factors for PPUI?	Advanced age, radiotherapy, larger prostate, and shorter membranous urethra increase risk. Surgical approach has little impact, except for preserving neurovascular bundles. BMI may affect short-term, but not long-term, continence.	Risk factors for incontinence include advanced age, high BMI, LUTS, poor baseline continence, short membranous urethra, weak sphincter or pelvic floor, large prostate, aggressive cancer, comorbidities, and prior pelvic surgery or radiation. **CORRECT**	Preoperative risk factors include advanced age, high BMI, LUTS, diabetes, weak pelvic floor muscles, and poor physical fitness. Surgical experience and technique also influence outcomes. Psychological factors like anxiety may affect recovery. **PARTIALLY CORRECT:***It doesn't mention urethral length or radiotherapy*
Question based on clinical case*			
A 66-year-old man with mild SUI, two years post-prostatectomy and unresponsive to conservative treatment, seeks surgery. He has no urgency and underwent salvage radiotherapy. Is a male sling appropriate?	Male slings should not be routinely performed in patients with severe stress incontinence.	No, a male sling is generally not the preferred surgical option for this patient due to his history of salvage radiotherapy. **CORRECT**	Yes, surgical treatment with a male sling is a reasonable option for this patient. **INCORRECT:***Considers a sling an option in this post-radiotherapy patient*

This table provides a comparison of responses from ChatGPT-4 and DeepSeek for selected conceptual and case-based questions related to PPUI, highlighting instances where their performance differed from guideline recommendations.

BMI = body mass index; LUTS = lower urinary tract symptoms; SUI = stress urinary incontinence; PPUI = postprostatectomy urinary incontinence.

* Questions, answers, and recommendations in this table are abbreviated

**Table 2:** Performance of ChatGPT and DeepSeek according to guideline domains.

Domain (number of questions)	ChatGPT			DeepSeek			
	Correct	Partial	Wrong		Correct	Partial	Wrong
Preprostate treatment (2)	2				1	1	
Postprostate treatment (3)	3				2		1
Evaluation (5)	4	1			3	2	
Treatment options (7)	6	1			4	2	1
Complications (2)	2				1	1	
Special situations (1)	1					1	
Total ^20^^a^	95.0% b			72.5% c			

This table summarizes the performance of ChatGPT and DeepSeek across various guideline domains, indicating the number of correct, partially correct, and incorrect responses for both conceptual and case-based questions.

a Ten conceptual and ten are case-based questions.

b Eighteen correct; two partially correct.

c Eleven correct; seven partially correct.

**Test 1:** In the conceptual questions, ChatGPT provided accurate answers to eight questions and partially correct answers to two, resulting in a final score of 9.0. No incorrect responses were recorded. In contrast, DeepSeek provided six correct answers and four partially correct responses, with a final score of 8.0 (p = 0.50).

**Test 2:** ChatGPT provided fully correct answers to all ten case-based questions, achieving a perfect score of 10.0. In contrast, DeepSeek provided five correct answers, three partially correct responses, and two incorrect answers, resulting in a final score of 6.5 (p = 0.08).

Tables 2 and 3 show the differences in performance between ChatGPT and DeepSeek, according to the different domains of the guideline. ChatGPT outperformed DeepSeek in all domains. Its two partially correct responses occurred in the *Evaluation* and *Treatment Options* domains. One of ChatGPT's partially correct answers was in response to a question about the need for urethrocystoscopy. It stated that the exam *should be considered* in patients being evaluated for surgical treatment of PPUI and correctly listed its principal utilities in this context; however, it did not classify the procedure as mandatory. The other partially correct response involved selecting patients with PPUI for sling placement. Although ChatGPT appropriately identified the incontinence as *mild to moderate* and noted the absence of prior pelvic radiotherapy, it failed to mention the time interval between surgery and treatment. It is considered mandatory in the preoperative evaluation.

**Table 3:** Wrong answers from ChatGPT and DeepSeek.

Guideline domains	ChatGPT	DeepSeek
**Pre-prostate treatment**		It does not mention urethral length or radiotherapy ^a^
Post-prostate treatment		Failed to recognize the ideal time to treat PPUI ^a^
Evaluation of PPUI	Did not consider cystoscopy mandatory in the preoperative evaluation^a^	Did not consider cystoscopy mandatory in the preoperative evaluation ^a^
		Considered urodynamic study as one of the mandatory tests in the preoperative evaluation ^a^
Treatment options	Does not mention the time from surgery to PPUI treatment; considers urodynamics mandatory in the preoperative evaluation ^a^	Considers urodynamics mandatory to confirm urethral mobility and function ^a, b^
		Offers a sling as a reasonable option for a man with a history of radiotherapy c
**Complications after PPUI surgery**		Considers urodynamics essential in a patient with clearly defined pure SUI based on history and physical exam; does not mention cistoscopy ^a^
Special situations		Fails to acknowledge the need to treat BNC prior to AUS implantation ^a^

This table details the specific errors made by ChatGPT and DeepSeek, categorized by guideline domain, including instances of partially correct and incorrect answers.

a Partially correct;

b An error was made in two different questions;

c Wrong answer.

PPUI = post-prostatectomy urinary incontinence; SUI = stress urinary incontinence; BNC = bladder neck contracture; AUS = artificial urinary sphincter.

DeepSeek presented partial or complete errors across all six guideline domains. Its poorest performance was in *Treatment Options*, where it provided two partially correct answers and one incorrect response. The other incorrect answer occurred in the *Pre-prostate Treatment* domain. Additionally, two partially correct answers were given in the *Evaluation* domain, and one partially correct response was observed in each of the following domains: *Pre-prostate TreatmentComplications*, and *Special Situations*

Neither chatbots informed users that their answers may be based on general training data rather than specific, up-to-date, high-quality medical content, nor did they provide warnings about the limitations of their training data or the potential for inaccuracies. In a few responses (one conceptual and two case-based), DeepSeek highlighted the importance of shared decision-making, noting that surgical treatment involves risks and requires realistic expectations regarding outcomes. It also recommended early referral to a specialist in male incontinence to optimize the timing and appropriateness of the intervention. ChatGPT did not include explicit recommendations to consult a physician or healthcare professional. Some responses advised that a thorough discussion of risks, benefits, and the potential need for lifelong device management is essential to support better outcomes.

## DISCUSSION

This study aimed to evaluate and compare the accuracy of two LLMs, ChatGPT-4o and DeepSeek R1, in generating guideline-concordant recommendations for the management of PPUI, using the AUA/SUFU guideline (12) as a benchmark. This direct, head-to-head comparison of these two contemporary LLMs in a specialized urological context provides novel insights into their relative clinical utility and potential safety implications. ChatGPT outperformed DeepSeek, achieving an overall accuracy of 95.0% versus 72.5%, respectively (p = 0.031). This performance gap was especially pronounced in case-based scenarios, where ChatGPT scored a perfect 10/10, while DeepSeek obtained 6.5/10. These results reinforce previous findings from our group (5) and from an independent study (13), where ChatGPT-4 significantly surpassed version 3.5 in similar clinical contexts.

ChatGPT demonstrated consistently high performance across all evaluated guideline domains, particularly in complex areas such as treatment selection and surgical complication management. In contrast, DeepSeek produced partially or fully incorrect responses in every domain, with its weakest performance in the "treatment options" section. Notably, many of these inaccuracies were critical rather than minor; for example, recommending a male sling for a radiated patient represents a potentially hazardous clinical decision, not a trivial factual slip, thereby heightening safety concerns. While DeepSeek occasionally incorporated ethical elements, such as promoting shared decision-making and specialist referral, these did not offset its factual inaccuracies. This divergence likely reflects differences in training architecture, particularly reinforcement learning with human feedback, which has been associated with improved factual reliability in newer LLMs (1)

Both models were tested under a zero-shot prompt configuration without prior context or instruction to follow a specific guideline. This design was intentional, simulating real-world interactions where users input spontaneous questions. Under these conditions, ChatGPT's strong contextual alignment with evidence-based recommendations suggests real-world utility in clinical support or educational use. Nonetheless, performance was not flawless. For instance, ChatGPT failed to state that cystoscopy is mandatory in the preoperative workup and incorrectly portrayed urodynamic testing as universally required, demonstrating that even high-performing LLMs may falter in nuanced or infrequently referenced scenarios.

The study methodology included domain-stratified question design by experienced urologists and structured, independent evaluation. Although the assessment framework was rigorous, the absence of a blinded review may have introduced bias. Additionally, as all prompts were in English, the findings may not generalize to other languages or cultural contexts.

ChatGPT's high accuracy in guideline-based PPUI scenarios supports its potential as a supplementary tool in urologic clinical practice. While not a substitute for clinical judgment, it may aid physicians in patient education, draft preparation, or as a second opinion generator. Similar performance advantages have been noted in other urologic conditions – including benign prostatic hyperplasia, prostate cancer, and urolithiasis – where ChatGPT-generated materials have been favorably rated compared to traditional resources (14)

These findings also underscore the broader potential of LLMs to improve healthcare delivery across diverse settings (15). In resource-limited environments, where access to trained specialists is constrained but digital connectivity is increasingly available, LLMs may serve as a valuable tool to support triage and immediate clinical decision-making (16). Beyond access, these models may enhance healthcare system efficiency by organizing and synthesizing complex clinical information, facilitating the monitoring of patients with multiple comorbidities, and reducing the administrative workload that often burdens clinicians (1)

Nonetheless, current LLMs lack key capabilities for individualized care. They do not reliably account for patient-specific variables such as comorbidities, psychosocial context, or long-term treatment trajectories. Moreover, neither model provided disclaimers regarding the scope or limitations of their training data, nor did they express uncertainty or confidence levels in their outputs. These omissions pose risks, particularly in unsupervised or low-resource environments. As static systems, LLMs that do not incorporate real-time updates may become outdated or misleading, further underscoring the need for continuous monitoring and recalibration (17)

The limitations observed in this study align with the broader literature on LLM performance in Medicine. Recent evaluations in pediatric urology have demonstrated that while ChatGPT offers general knowledge, its responses can be incomplete, ambiguous, and even misleading, particularly when compared to formal guidelines for conditions like phimosis (18). While ChatGPT generally outperforms models such as Gemini and Bard in urologic domains, all tend to simplify or misrepresent complex clinical cases (4 6). Moreover, our comparison involved the premium version of ChatGPT and the free version of DeepSeek, which may have influenced the overall performance gap. Another limitation is the potential language bias, as both AI models were evaluated exclusively in English. DeepSeek's performance may differ – and possibly improve – in Chinese, the language in which it was originally developed and likely optimized. Future studies should explore multilingual evaluations, particularly in Chinese, to more accurately assess each model's full capabilities and real-world applicability across diverse linguistic contexts.

Another significant limitation is the relatively small sample size, which utilizes only 20 questions to assess model performance. Although the questions were carefully developed based on established clinical guidelines and reviewed by experienced urologists, this limited number may not fully represent the wide spectrum of real-world clinical decision-making scenarios. Small differences in interpretation or phrasing could disproportionately influence the results.

## CONCLUSIONS

While ChatGPT demonstrates superior performance over DeepSeek in the context of PPUI, both models currently lack the ability to autonomously guide clinical decision-making. Our findings support the view that AI can effectively augment — but not replace — physician expertise in Urology, highlighting the need for transparent, continuously updated, and supervised integration of LLMs into clinical workflows to ensure their safe and effective use.

## APPENDIX

Feb 3^rd^, 2025

**Table:** QUESTIONS WITH ANSWERS

**Question 1**	**What are the known preoperative risk factors that could affect the chance of developing urinary incontinence in patients undergoing radical prostatectomy? Your answer must be in one paragraph and a maximum of 200 words.**
**GPT 4o** **1,0**	Preoperative risk factors for urinary incontinence following radical prostatectomy include advanced age, higher body mass index (BMI), preexisting lower urinary tract symptoms (LUTS), and poor baseline continence. Anatomical and functional factors such as a shorter membranous urethral length, decreased urethral sphincter function, and reduced pelvic floor muscle strength also contribute. Larger prostate volume and more aggressive prostate cancer (high Gleason score, extracapsular extension) may necessitate extensive dissection, increasing the risk of sphincter damage. Comorbidities like diabetes mellitus, cardiovascular disease, and neurological disorders can impair nerve and muscle function, further predisposing patients to postoperative incontinence. Additionally, prior pelvic surgeries or radiation therapy may compromise sphincter integrity and urethral support. Understanding these risk factors is crucial for patient counseling and prehabilitation strategies, such as pelvic floor muscle training before surgery, to improve postoperative outcomes.
**DeepSeek** **0,5**	Several preoperative risk factors have been identified that may increase the likelihood of developing urinary incontinence (UI) following radical prostatectomy. Advanced age is a significant predictor, as older patients often have reduced pelvic muscle tone and slower recovery. Higher body mass index (BMI) is also associated with increased UI risk due to added pressure on the pelvic floor. Pre-existing lower urinary tract symptoms (LUTS), such as urgency or weak stream, can exacerbate postoperative incontinence. Additionally, a history of diabetes or other conditions affecting nerve function may impair bladder control. Surgical factors, such as the surgeon's experience and the surgical technique used (e.g., nerve-sparing vs. non-nerve-sparing), also play a role, though these are not strictly preoperative. Preoperative pelvic floor muscle strength and the patient's overall physical fitness level can influence recovery, with weaker muscles correlating with higher UI risk. Lastly, psychological factors, such as anxiety or depression, may indirectly affect outcomes by imp acting adherence to rehabilitation exercises. Identifying and addressing these risk factors preoperatively can help tailor interventions to mitigate the risk of UI post-surgery.
**Question 2**	**In patients undergoing radical prostatectomy, is incontinence expected in the short-term following surgery? Your answer must be in one paragraph and a maximum of 200 words.**
**GPT 4o** **1,0**	Yes, urinary incontinence is expected in the short-term following radical prostatectomy, primarily due to the disruption of the anatomical structures responsible for continence, including the external urethral sphincter and supportive pelvic tissues. The immediate postoperative period often involves stress urinary incontinence (SUI), characterized by leakage during activities that increase intra-abdominal pressure. Contributing factors include sphincteric dysfunction, loss of urethral length, and temporary neuropraxia affecting the pelvic floor muscles. Most patients experience significant improvement within the first year as sphincter function recovers and pelvic floor muscles strengthen. Early recovery is influenced by preoperative continence status, surgical technique (e.g., nerve-sparing approaches), and adherence to pelvic floor muscle training. While the majority regain continence within 6 to 12 months, a subset may have persistent incontinence requiring additional interventions such as pelvic floor therapy, pharmacologic treatment, or surgical management with slings or artificial urinary sphincters.
**DeepSeek** **1,0**	Yes, incontinence is a common short-term complication following radical prostatectomy, affecting a significant proportion of patients initially. The surgery involves removing the prostate gland, which can disrupt the urinary sphincter mechanism and surrounding structures responsible for urinary control. In the immediate postoperative period, many patients experience stress urinary incontinence (leakage with coughing, sneezing, or exertion) or urge incontinence (sudden, uncontrollable need to urinate). Studies suggest that up to 50-80% of patients may experience some degree of incontinence shortly after surgery, though the severity varies. Pelvic floor muscle exercises, behavioral modifications, and time often lead to gradual improvement, with many patients regaining continence within 3 to 12 months. However, a small percentage may continue to experience persistent incontinence, necessitating further interventions such as physical therapy, medications, or surgical options like artificial urinary sphincter placement. Factors such as age, surgical technique, preoperative urinary function, and surgeon experience can influence the likelihood and duration of incontinence. Patients should be counseled preoperatively about this risk and the expected recovery trajectory to manage expectations and encourage adherence to postoperative rehabilitation strategies.
**Question 3**	**In patients who have undergone radical prostatectomy, do pelvic floor muscle exercises or pelvic floor muscle training help recovering urinary continence in the early post-operative period? Your answer must be in one paragraph and a maximum of 200 words.**
**GPT 4o** **1,0**	Yes, pelvic floor muscle exercises (PFME) or pelvic floor muscle training (PFMT) can aid in the recovery of urinary continence in the early post-operative period after radical prostatectomy. Studies have shown that structured PFMT improves continence rates and shortens the duration of post-prostatectomy incontinence by enhancing the strength and coordination of the pelvic floor muscles, which compensate for the loss of urethral sphincter function. Early initiation, ideally preoperatively or within the first few week's post-surgery, appears to be beneficial in accelerating continence recovery. Supervised programs by physiotherapists or trained specialists tend to yield better outcomes compared to unsupervised exercises. However, the magnitude of benefit varies, and some patients may continue to experience incontinence despite training. While PFMT is generally recommended as a first-line conservative approach, its efficacy should be evaluated in the context of individual patient factors, including baseline pelvic floor function and adherence to the exercise regimen.
**DeepSeek** **1,0**	Yes, pelvic floor muscle exercises (PFME) or pelvic floor muscle training (PFMT) have been shown to aid in the recovery of urinary continence in the early post-operative period following radical prostatectomy. Studies indicate that structured PFMT programs, when initiated soon after catheter removal, can significantly improve the rate and speed of regaining urinary control compared to no intervention or general advice alone. These exercises strengthen the pelvic floor muscles, which are often weakened or damaged during surgery, thereby enhancing urethral sphincter function and reducing urinary leakage. Early and consistent PFMT, often guided by a physiotherapist, helps patients regain continence more quickly, with some evidence suggesting improved outcomes within the first 3-6 months post-surgery. However, the extent of benefit may vary based on individual factors such as preoperative continence status, surgical technique, and adherence to the exercise regimen. While PFMT is generally recommended as a non-invasive and effective approach, it is often most beneficial when combined with other strategies like behavioral modifications and patient education. Overall, early initiation of PFMT is a valuable component of post-prostatectomy rehabilitation for improving urinary continence outcomes.
**Question 4**	**In patients with bothersome stress urinary incontinence after radical prostatectomy, when should surgical treatment be offered? Your answer must be in one paragraph and a maximum of 200 words.**
**GPT 4o** **1,0**	Surgical treatment for bothersome stress urinary incontinence (SUI) after radical prostatectomy should be considered at least **12 months postoperatively** to allow for maximal spontaneous recovery of continence. However, earlier intervention may be appropriate in cases of severe incontinence significantly impacting quality of life. Before surgery, patients should undergo a thorough evaluation, including **urodynamic studies and cystoscopy**, to assess sphincter function, bladder compliance, and rule out anastomotic strictures or detrusor dysfunction. Conservative measures, such as **pelvic floor muscle training and behavioral modifications**, should be optimized before proceeding with surgery. The **artificial urinary sphincter (AUS)** remains the gold standard for moderate to severe SUI, while **male slings** are an option for patients with mild to moderate incontinence and adequate sphincter function. Patient preference, comorbidities, and prior treatments should guide decision-making.
**DeepSeek** **1,0**	Surgical treatment for bothersome stress urinary incontinence (SUI) after radical prostatectomy should be considered after a thorough evaluation and a period of conservative management. Typically, surgical intervention is offered if significant incontinence persists beyond 6 to 12 months post-surgery, as spontaneous improvement may occur during this time. Prior to considering surgery, patients should undergo a comprehensive assessment, including urodynamic studies, to confirm the diagnosis of SUI and rule out other causes of incontinence, such as detrusor overactivity or bladder outlet obstruction. Conservative measures, including pelvic floor muscle training, lifestyle modifications, and possibly pharmacotherapy, should be attempted first. If these measures fail to provide adequate symptom relief and the patient's quality of life remains significantly impacted, surgical options such as an artificial urinary sphincter (AUS) or male sling can be considered. The choice of procedure depends on factors such as the severity of incontinence, patient preferences, and surgeon expertise. Shared decision-making is essential, as surgical treatment carries risks and requires realistic expectations regarding outcomes. Early referral to a specialist in male incontinence is recommended to optimize timing and appropriateness of intervention.
**Question 5**	**In patients being considered for surgical treatment for stress urinary incontinence following radical prostatectomy, should cystourethroscopy be performed? Your answer must be in one paragraph and a maximum of 200 words.**
**GPT 4o** **0,5**	Yes, cystourethroscopy should be considered in patients being evaluated for surgical treatment of stress urinary incontinence (SUI) after radical prostatectomy. This examination helps assess urethral integrity, the presence of anastomotic strictures, bladder neck contractures, or residual sphincteric function, which can impact surgical planning and outcomes. Identifying urethral pathology preoperatively allows for appropriate management, such as treating strictures before artificial urinary sphincter (AUS) implantation or selecting an alternative surgical approach. Additionally, cystourethroscopy can help determine whether there is significant bladder dysfunction, which may affect the choice between an AUS and a male sling. While not always mandatory in straightforward cases, it provides valuable anatomical and functional insights that contribute to optimal surgical decision-making and improved patient outcomes.
**DeepSeek** **0,5**	In patients being considered for surgical treatment for stress urinary incontinence (SUI) following radical prostatectomy, cystourethroscopy is generally recommended as part of the preoperative evaluation. This procedure allows for the direct visualization of the lower urinary tract, helping to identify potential anatomical abnormalities, urethral strictures, bladder neck contractures, or other pathologies that could contribute to incontinence or affect surgical planning. Additionally, cystourethroscopy can assess the integrity of the urethral sphincter and bladder neck, which are critical for determining the most appropriate surgical intervention, such as an artificial urinary sphincter or male sling. It also helps rule out other causes of urinary symptoms, such as bladder stones or tumors, which may require alternative management. While cystourethroscopy is not mandatory in all cases, it provides valuable diagnostic information that can optimize patient selection, improve surgical outcomes, and reduce the risk of complications. Therefore, it is considered a useful and often necessary component of the preoperative workup for post-prostatectomy SUI.
**Question 6**	**In patients being considered for surgical treatment for stress urinary incontinence following radical prostatectomy, should urodynamic testing be performed? Your answer must be in one paragraph and a maximum of 200 words.**
**GPT 4o** **1,0**	Yes, urodynamic testing should be considered in patients undergoing surgical treatment for stress urinary incontinence (SUI) following radical prostatectomy, though its routine use remains debated. Urodynamics can help differentiate between true sphincteric insufficiency and other contributing factors such as detrusor overactivity, reduced bladder compliance, or impaired bladder contractility, which may influence treatment decisions. Identifying detrusor overactivity may prompt consideration of conservative or pharmacologic management before proceeding with surgery. Additionally, confirming adequate bladder contractility is crucial, particularly in patients at risk for post-surgical voiding dysfunction, such as those with detrusor underactivity. However, some studies suggest that clinical evaluation alone, including history, physical examination, and pad test, may be sufficient in straightforward cases, as urodynamic findings rarely alter surgical planning when intrinsic sphincter deficiency is the predominant cause. Therefore, while not mandatory in all cases, urodynamic testing is particularly useful in patients with atypical symptoms, prior radiotherapy, previous incontinence surgeries, or suspected concomitant bladder dysfunction, helping to optimize patient selection and improve surgical outcomes.
**DeepSeek** **1,0**	In patients being considered for surgical treatment for stress urinary incontinence (SUI) following radical prostatectomy, urodynamic testing (UDS) is not universally mandatory but can provide valuable insights in specific cases. While clinical evaluation, including history, physical examination, and bladder diaries, often suffices to diagnose SUI, UDS may be warranted when the diagnosis is unclear, when mixed urinary symptoms (e.g., urgency or obstruction) are present, or when prior surgical interventions have failed. UDS helps differentiate between intrinsic sphincter deficiency (the primary cause of post-prostatectomy SUI) and other contributing factors, such as detrusor overactivity or bladder outlet obstruction, which could influence surgical planning and outcomes. However, routine use of UDS in straightforward cases of SUI may not significantly alter management or improve results, as surgical decisions are often based on clinical findings. Therefore, UDS should be selectively employed based on individual patient complexity and the need for additional diagnostic clarity to optimize treatment strategies.
**Question 7**	**What is the ideal patient profile for the implantation of an artificial urinary sphincter in men with stress urinary incontinence after radical prostatectomy seeking for surgical treatment? Your answer must be in one paragraph and a maximum of 200 words.**
**GPT 4o** **1,0**	The ideal candidate for artificial urinary sphincter (AUS) implantation in men with stress urinary incontinence (SUI) following radical prostatectomy is one with moderate to severe SUI persisting for at least 12 months despite conservative measures, such as pelvic floor muscle training. He should have good cognitive function and manual dexterity to operate the device, or a reliable caregiver if assistance is needed. A stable bladder with adequate capacity, compliance, and the absence of detrusor overactivity or significant post-void residual volume is crucial to prevent complications. Patients should have no evidence of active infection, urethral stricture, bladder neck contracture, or prior pelvic radiation with severe fibrosis, as these conditions increase the risk of erosion and device failure. Good general health, with the ability to tolerate anesthesia and surgery, is also important. Psychological readiness and realistic expectations regarding potential complications, device longevity, and the need for future revisions should be assessed preoperatively. Proper patient selection optimizes outcomes, ensuring satisfactory continence restoration and quality-of-life improvement.
**DeepSeek** **0,5**	The ideal patient profile for the implantation of an artificial urinary sphincter (AUS) in men with stress urinary incontinence (SUI) after radical prostatectomy includes individuals with persistent, bothersome SUI that significantly impacts their quality of life, typically after a minimum of 6-12 months of conservative management (e.g., pelvic floor exercises) without sufficient improvement. Candidates should have adequate manual dexterity and cognitive function to operate the device, as well as realistic expectations regarding outcomes, including the potential need for revisions or explantation. They should be in good overall health with no active urinary tract infections, untreated bladder dysfunction, or severe detrusor overactivity, as these conditions may compromise surgical success. Urodynamic studies are often recommended to confirm SUI and rule out other contributing factors. Patients with prior radiation therapy or urethral strictures may still be candidates but require careful evaluation due to higher risks of complications. A thorough assessment of urethral integrity and bladder capacity is essential. The AUS is particularly suitable for patients seeking a durable, effective solution for SUI who are willing to accept the risks of mechanical failure, infection, or erosion, and who are committed to long-term follow-up.
**Question 8**	**What is the ideal patient profile for the implantation of sling in men with stress urinary incontinence after radical prostatectomy seeking for surgical treatment? Your answer must be in one paragraph and a maximum of 200 words.**
**GPT 4o** **0,5**	The ideal candidate for male sling implantation to treat stress urinary incontinence (SUI) after radical prostatectomy is a patient with mild to moderate incontinence, typically defined as requiring ≤3 pads per day, with preserved or mildly compromised detrusor contractility. Candidates should have an intact external urethral sphincter without significant fibrosis or scarring, confirmed through clinical evaluation and urethroscopy. The patient should not have a history of pelvic radiation therapy or previous urethral surgery, as these conditions increase the risk of failure. Urodynamic studies should demonstrate adequate bladder compliance and the absence of significant detrusor overactivity. The ideal candidate is motivated, has realistic expectations regarding surgical outcomes, and understands that slings are less effective for severe incontinence compared to artificial urinary sphincters.
**DeepSeek** **0,5**	The ideal patient profile for male sling implantation in post-radical prostatectomy stress urinary incontinence (SUI) includes men with mild to moderate incontinence (typically defined as 1-2 pads per day), who have failed or declined conservative management such as pelvic floor exercises or behavioral therapy. Candidates should have a stable or improved urinary condition for at least 6-12 months post-prostatectomy, ensuring no further recovery of continence is expected. They should have adequate urethral mobility and function, confirmed through urodynamic studies, and no significant detrusor overactivity or reduced bladder compliance. Additionally, patients should be in good general health, without untreated bladder outlet obstruction, recurrent urethral strictures, or prior radiation therapy, as these factors can compromise outcomes. Realistic expectations regarding the potential for improvement rather than complete continence are essential, as sling procedures aim to reduce leakage rather than fully restore pre-surgery function. A thorough evaluation, including pad tests, cystoscopy, and patient-reported symptom severity, is crucial to confirm suitability for the procedure.
**Question 9**	**In men with stress urinary incontinence after primary, adjuvant, or salvage radiotherapy who are seeking surgical management, what is the most appropriate treatment alternative? Your answer must be in one paragraph and a maximum of 200 words.**
**GPT 4o** **1,0**	In men with stress urinary incontinence (SUI) following primary, adjuvant, or salvage radiotherapy, the artificial urinary sphincter (AUS) remains the gold-standard surgical treatment due to its proven efficacy in restoring continence. Radiotherapy-induced fibrosis and tissue atrophy increase the risk of complications, such as urethral atrophy and erosion, making AUS a preferred choice over male slings, which require preserved urethral integrity and sufficient sphincteric function. In select cases of mild-to-moderate SUI with adequate urethral compliance, an adjustable balloon device may be considered as an alternative, offering modulation of continence control postoperatively. However, the risk of infection, erosion, and device malfunction is higher in radiated patients, necessitating careful patient selection and counseling. Multidisciplinary evaluation, including urodynamic studies and endoscopic assessment, is crucial to guide surgical planning.
**DeepSeek** **1,0**	In men with stress urinary incontinence (SUI) following primary, adjuvant, or salvage radiotherapy, the most appropriate surgical treatment is typically the implantation of an artificial urinary sphincter (AUS). The AUS is considered the gold standard for managing moderate to severe SUI in this population due to its high efficacy and durability. It consists of a cuff placed around the bulbar urethra, a pressure-regulating balloon in the retroperitoneum, and a pump in the scrotum, allowing the patient to manually control urination. While other options, such as male slings, may be considered for mild to moderate SUI, they are generally less effective in post-radiation patients due to compromised tissue integrity and reduced urethral mobility caused by radiation-induced fibrosis. The AUS, however, is better suited to address the complex pathophysiology of SUI in this context. Careful patient selection, preoperative counseling, and management of expectations are crucial, as complications such as infection, erosion, or mechanical failure can occur. Despite these risks, the AUS offers significant improvements in quality of life and continence rates, making it the preferred surgical option for post-radiation SUI in men.
**Question 10**	**Prior to implantation of an artificial urinary sphincter as surgery for stress urinary incontinence after radical prostatectomy, what should clinicians evaluate to ensure that patients will have the ability to operate the device? Your answer must be in one paragraph and a maximum of 200 words.**
**GPT 4o** **1,0**	Before implanting an artificial urinary sphincter (AUS) for stress urinary incontinence following radical prostatectomy, clinicians must evaluate the patient's cognitive function, manual dexterity, and motivation to ensure they can properly operate the device. Cognitive assessment is crucial to confirm the patient understands how to manipulate the control mechanism and adhere to postoperative care instructions. Manual dexterity must be sufficient to locate, squeeze, and release the control pump, typically positioned in the scrotum. Conditions such as arthritis, neuropathy, or prior hand surgery should be considered. Additionally, assessing the patient's motivation and expectations is essential, as successful use requires active participation in device operation. Urodynamic studies should confirm adequate bladder compliance and capacity, ruling out detrusor overactivity or reduced compliance that could compromise AUS function. A thorough discussion of the risks, benefits, and potential need for lifelong device management is necessary to ensure patient commitment
**DeepSeek** **1,0**	Prior to implantation of an artificial urinary sphincter (AUS) for stress urinary incontinence following radical prostatectomy, clinicians must evaluate the patient's physical and cognitive ability to operate the device effectively. This includes assessing manual dexterity, as the device requires manipulation of a scrotal or labial pump to control urine flow. Patients with conditions such as arthritis, neuropathy, or other impairments affecting hand strength or coordination may struggle to use the device. Cognitive function should also be evaluated to ensure the patient can understand and follow the necessary steps to operate the AUS, particularly in cases of dementia or other cognitive disorders. Additionally, clinicians should consider the patient's motivation and willingness to comply with the device's operation and maintenance. A thorough discussion of the device's mechanism, potential complications, and the patient's expectations is essential to confirm their ability and commitment to using the AUS. If the patient is deemed unable to operate the device, alternative treatments for incontinence should be explored. This comprehensive evaluation ensures the AUS is a suitable and effective option for the patient's specific needs.
**Question 11**	**A 65-year-old male patient who is bothered with severe stress urinary incontinence after radical prostatectomy and considering surgical treatment presents for preoperative evaluation. The patient has no complaints of urgency or urgeincontinence, has a good urinary flow and stress urinary incontinence is demonstrated on physical examination. What further tests are needed before surgery? Your answer must be in one paragraph and a maximum of 200 words.**
**GPT 4o** **1,0.**	In the preoperative evaluation of this 65-year-old male with severe stress urinary incontinence (SUI) after radical prostatectomy, key additional tests are needed to optimize surgical planning. A **24-hour pad test** or **pad weight test** quantifies urine loss severity. **Uroflowmetry** and **post-void residual (PVR) measurement** confirm adequate emptying. **Cystoscopy** assesses urethral integrity, the presence of anastomotic strictures, and bladder neck competence. **Urodynamic testing** (optional but recommended in select cases) helps rule out detrusor dysfunction, impaired compliance, or occult detrusor overactivity, especially if prior radiation therapy was received. A **videourodynamic study** may be particularly useful if there is concern about bladder dysfunction. Finally, assessment of **manual dexterity and cognitive function** ensures the patient can manipulate a device like an artificial urinary sphincter (AUS), if chosen. These evaluations guide surgical decision-making, ensuring appropriate treatment selection and minimizing postoperative complications.
**DeepSeek** **0,5**	Before proceeding with surgical treatment for stress urinary incontinence (SUI) post-radical prostatectomy, further evaluation should include urodynamic studies to confirm the diagnosis of pure SUI, assess bladder capacity, compliance, and detrusor function, and rule out detrusor overactivity or poor compliance that could affect surgical outcomes. A 24-hour pad test can quantify the severity of incontinence, while cystoscopy may be performed to evaluate the urethral sphincter integrity, bladder neck, and urethral anatomy, ensuring no obstruction or other abnormalities. Post-void residual urine measurement should be done to confirm complete bladder emptying. Additionally, a urinalysis and urine culture are necessary to exclude infection. If the patient has a history of radiation therapy or complex pelvic surgery, imaging such as a retrograde urethrogram or MRI may be considered to assess tissue quality and anatomy. These tests help ensure the patient is an appropriate candidate for surgery, such as an artificial urinary sphincter or male sling, and optimize the likelihood of a successful outcome.
**Question 12**	**A 78-year-old male patient who is bothered with severe stress urinary incontinence after radical prostatectomy and considering surgical treatment presents for preoperative evaluation. The patient has Parkinson's disease and complains of both stress and urgeincontinence. He has a good urinary flow and stress urinary incontinence is demonstrated on physical examination. Cystourethroscopy is normal. Should other tests be obtained before surgery? Your answer must be in one paragraph and a maximum of 200 words.**
**GPT 4o** **1,0**	Yes, additional tests should be obtained before proceeding with surgery, given the patient's Parkinson's disease and mixed urinary incontinence (stress and urge incontinence). A urodynamic study is essential to assess detrusor function, given the high likelihood of detrusor overactivity or impaired compliance in neurogenic conditions, which could impact surgical outcomes. If significant detrusor overactivity or poor bladder compliance is present, stress incontinence might not be the sole contributor to his symptoms, and surgery alone may not provide satisfactory results. Additionally, a post-void residual (PVR) measurement should be performed to evaluate potential bladder dysfunction, especially given his neurological condition. If PVR is high, this may indicate underlying detrusor underactivity, which could lead to postoperative urinary retention. Finally, a careful neurological assessment should be conducted to evaluate the severity of Parkinson's disease, as advanced disease may increase the risk of postoperative complications and affect the patient's ability to manage a device such as an artificial urinary sphincter. These evaluations will help guide surgical decision-making and optimize outcomes.
**DeepSeek** **1,0**	Before proceeding with surgical treatment for stress urinary incontinence (SUI) in this patient, additional tests are warranted to ensure an accurate diagnosis and appropriate management. Given his Parkinson's disease, which can contribute to both stress and urge incontinence due to detrusor overactivity and impaired bladder control, a urodynamic study (UDS) is essential. UDS will help differentiate between the components of his incontinence, assess bladder capacity, detrusor function, and urethral sphincter competence, and rule out detrusor overactivity or poor bladder compliance, which could influence surgical outcomes. Additionally, a 24-hour pad test can quantify the severity of incontinence, providing objective data to guide treatment decisions. Since he has a good urinary flow and normal cystourethroscopy, obstruction is unlikely, but UDS will confirm this. Given his age and Parkinson's disease, a comprehensive geriatric assessment may also be beneficial to evaluate his overall fitness for surgery and identify any factors that could impact postoperative recovery. These tests will ensure that the chosen surgical intervention, such as an artificial urinary sphincter or male sling, is appropriate and likely to provide optimal results while minimizing risks.
**Question 13**	**A 67-year-old male patient with severe stress urinary incontinence persisting 3 months after radical prostatectomy, who have already undergone pelvic floor muscle exercises, desires surgical treatment for his condition. Should the implantation of an artificial urinary sphincter be considered in this scenario? You answer must be in one paragraph and a maximum of 200 words.**
**GPT 4o** **1,0**	No, the implantation of an artificial urinary sphincter (AUS) should not be immediately considered in this scenario, as severe stress urinary incontinence (SUI) persisting at only three months post-radical prostatectomy is still within the expected recovery period. While early severe SUI may predict long-term incontinence, conservative management, including pelvic floor muscle training, is typically continued for at least 6 to 12 months before considering surgical intervention. Most guidelines recommend waiting at least 6 months post-prostatectomy to allow for potential spontaneous improvement. However, in select cases of severe, bothersome incontinence with minimal improvement over time, earlier intervention may be considered, particularly if quality of life is significantly impacted. If symptoms persist beyond 6–12 months, the patient could be evaluated for AUS implantation, which remains the gold standard for moderate-to-severe post-prostatectomy incontinence. A thorough assessment, including urodynamics if necessary, should confirm sphincteric deficiency without bladder dysfunction before proceeding.
**DeepSeek** **0**	Yes, the implantation of an artificial urinary sphincter (AUS) should be considered in this scenario. AUS is a well-established and effective surgical treatment for severe stress urinary incontinence (SUI) in men, particularly after radical prostatectomy when conservative measures like pelvic floor muscle exercises have failed. The device, typically placed around the bulbar urethra, allows the patient to manually control urination by inflating or deflating a cuff. Studies have shown significant improvement in continence rates and quality of life for patients with post-prostatectomy SUI who undergo AUS implantation. However, the decision should be made after a thorough evaluation, including urodynamic studies to confirm SUI severity and exclude other causes of incontinence. The patient should also be informed about potential risks, such as infection, erosion, or mechanical failure, and the need for possible future revisions. Given the patient's age and persistent symptoms, AUS remains a viable and often preferred option for achieving long-term continence.
**Question 14**	**A 63-year-old male patient with urinary incontinence two years after radical prostatectomy, presents with lower urinary tract symptoms characterized predominantly by urgency and urgeincontinence. He complains of occasional stress urinary incontinence when coughing or sneezing. He has a normal urinary flow, low post-void residual volume and a normal urinalysis. Should surgical treatment with a male sling be offered? Your answer must be in one paragraph and a maximum of 200 words.**
**GPT 4o** **1,0**	No, surgical treatment with a male sling is not the best option in this case. Male slings are typically indicated for mild to moderate stress urinary incontinence (SUI) with preserved sphincter function, but this patient's predominant symptoms are urgency and urge incontinence, which suggest detrusor overactivity rather than significant sphincteric deficiency. The occasional stress incontinence episodes do not justify a male sling, as it would not address his primary complaint. Given his normal urinary flow, low post-void residual volume, and absence of infection, further evaluation with urodynamic studies may be warranted to confirm detrusor overactivity. First-line treatment should focus on behavioral modifications and pharmacologic therapy with antimuscarinics or β3-agonists. If symptoms persist despite conservative management, advanced therapies such as intradetrusor botulinum toxin injection or neuromodulation may be considered.
**DeepSeek** **1,0**	In this 63-year-old male with mixed urinary incontinence (predominantly urge incontinence with occasional stress incontinence) two years post-radical prostatectomy, surgical treatment with a male sling may be considered but is not the first-line option. His symptoms suggest a combination of detrusor overactivity (urgency, urge incontinence) and mild stress urinary incontinence (SUI). Given his normal urinary flow, low post-void residual volume, and normal urinalysis, there is no evidence of obstruction or infection. Initial management should focus on behavioral modifications, pelvic floor muscle training, and pharmacologic therapy (e.g., anticholinergics or beta-3 agonists) for urge incontinence. If SUI persists and significantly impacts his quality of life after conservative measures, a male sling or artificial urinary sphincter could be considered. However, the male sling is typically more effective for mild to moderate SUI, and its success may be limited if urge symptoms remain predominant. A thorough urodynamic evaluation is recommended to confirm the diagnosis and guide treatment. Shared decision-making is essential, weighing the risks and benefits of surgery against the patient's symptoms and preferences.
**Question 15**	**A 66-year-old male patient with stress urinary incontinence two years after radical prostatectomy, and who failed conservative treatments for his condition, presents for surgical treatment. His stress urinary incontinence is mild and he has no complaints of urgency or urgeincontinence. His past medical history includes salvage radiotherapy for biochemical prostate cancer recurrence. Should surgical treatment with a male sling be offered? Your answer must be in one paragraph and a maximum of 200 words.**
**GPT 4o** **1,0**	No, a male sling is generally not the preferred surgical option for this patient due to his history of salvage radiotherapy. While a male sling can be effective for mild stress urinary incontinence (SUI), its success is significantly lower in patients with prior pelvic radiation. Salvage radiotherapy can cause fibrosis, compromising urethral compliance and increasing the risk of failure or complications, such as urethral erosion. The artificial urinary sphincter (AUS) remains the gold standard for post-prostatectomy SUI, especially in radiated patients, as it provides better long-term outcomes and continence rates. However, given his mild incontinence, a thorough discussion should be conducted to assess the risks and benefits of AUS implantation versus ongoing conservative management, particularly considering the potential for worsened outcomes due to radiation-related tissue changes.
**DeepSeek** **0,0**	Yes, surgical treatment with a male sling is a reasonable option for this patient. The male sling is particularly suitable for patients with mild to moderate stress urinary incontinence (SUI) following radical prostatectomy, as it provides a less invasive alternative to an artificial urinary sphincter (AUS) with potentially fewer complications. Given that the patient has failed conservative treatments and has mild SUI without urgency or urge incontinence, he fits the profile of a good candidate for this procedure. The male sling works by compressing the bulbar urethra to improve continence, and studies have shown favorable outcomes in appropriately selected patients. However, the patient's history of salvage radiotherapy should be considered, as radiation can affect tissue quality and healing, potentially impacting surgical outcomes. While the male sling is generally effective, the patient should be counseled about the possibility of needing additional interventions, such as an AUS, if the sling does not provide adequate relief. Shared decision-making is essential, weighing the benefits, risks, and patient preferences. Overall, the male sling is a viable and appropriate surgical option for this patient.
**Question 16**	**A 79-year-old male patient with bothersome severe stress urinary incontinence five years after radical prostatectomy presents for the medical visit accompanied by his wife interested in surgical treatment. He uses more than 6 pads/day, and his urodynamics confirms pure stress urinary incontinence. He is under investigation with a neurologist for a progressive complaint of memory loss. Should surgical treatment with an artificial urinary sphincter be offered? Your answer must be in one paragraph and a maximum of 200 words.**
**GPT 4o** **1,0**	No, surgical treatment with an artificial urinary sphincter (AUS) should be approached with caution in this patient due to his ongoing evaluation for progressive memory loss. While he has severe stress urinary incontinence (SUI) confirmed by urodynamics and is highly symptomatic, cognitive decline can significantly impact his ability to understand, operate, and comply with the AUS mechanism, increasing the risk of device-related complications and failure. The decision should involve a thorough assessment by his neurologist to determine the extent and progression of cognitive impairment. If his cognitive function is preserved or the decline is minimal, AUS implantation may still be considered with strong caregiver support, particularly from his wife. However, if significant cognitive impairment is confirmed, alternative approaches such as absorbent products or, in select cases, a male sling may be more appropriate. A shared decision-making process, including discussions about the risks and long-term management, is essential.
**DeepSeek** **1,0**	In this case, the 79-year-old male patient with severe stress urinary incontinence (SUI) following radical prostatectomy is a potential candidate for an artificial urinary sphincter (AUS), given his significant symptom burden (≥6 pads/day) and urodynamic confirmation of pure SUI. However, his progressive memory loss, currently under neurological investigation, raises concerns about his ability to manage the device postoperatively, as AUS requires manual dexterity and cognitive understanding to operate the pump mechanism. A thorough assessment of his cognitive function and manual dexterity, along with caregiver support (e.g., his wife's involvement), is essential before proceeding. If he is deemed unable to operate the device independently, alternative treatments such as a male sling or continued conservative management may be more appropriate. Shared decision-making with the patient and his wife is crucial, weighing the benefits of improved continence against the risks and complexities of AUS implantation in the context of his cognitive decline.
**Question 17**	**A 81-year-old male patient with bothersome severe stress urinary incontinence five years after radical prostatectomy presents in consideration for surgical treatment. He uses more than 8 pads/day, and his urodynamics confirms pure stress urinary incontinence. His past medical history is significant for rheumatoid arthritis which significantly hampers his manual dexterity. Should surgical treatment with an artificial urinary sphincter be offered? Your answer must be in one paragraph and a maximum of 200 words.**
**GPT 4o** **1,0**	Yes, surgical treatment with an artificial urinary sphincter (AUS) can be considered for this patient, given his severe stress urinary incontinence (SUI) significantly affecting his quality of life. AUS is the gold standard for post-prostatectomy SUI, particularly in patients with high pad usage and confirmed urodynamic stress incontinence. However, his rheumatoid arthritis, which severely limits manual dexterity, raises concerns about his ability to manipulate the AUS activation mechanism. Alternative strategies include selecting a larger, easier-to-operate control pump, involving a caregiver for device activation, or considering alternative treatments such as male slings, though their efficacy is lower in severe SUI. A thorough preoperative discussion is essential to assess the patient's ability to use the device effectively and to explore support systems. If he cannot reliably operate the AUS, surgical intervention may not be beneficial, and other options, including external devices or catheterization, should be discussed.
**DeepSeek** **1,0**	Yes, surgical treatment with an artificial urinary sphincter (AUS) should be offered to this patient. He has severe stress urinary incontinence (SUI) following radical prostatectomy, significantly impacting his quality of life, as evidenced by his use of more than 8 pads/day. Urodynamic studies confirming pure SUI support the diagnosis and appropriateness of AUS, which is the gold standard for managing severe SUI in men post-prostatectomy. While his rheumatoid arthritis may complicate manual dexterity, the AUS can still be a viable option with proper patient education and support for device management. Alternative treatments, such as male slings, are less effective for severe incontinence, and bulking agents offer only temporary relief. Preoperative counseling should address the need for manual dexterity to operate the AUS and involve caregivers if necessary. Given the patient's age and medical history, a thorough evaluation of surgical risks and benefits is essential, but the AUS remains the most effective long-term solution for his condition.
Question 18	**A 67-year-old male patient with stress urinary incontinence two years after radical prostatectomy, and who failed conservative treatments for his condition, presents for surgical treatment. His stress urinary incontinence is severe and he has no complaints of urgency or urgeincontinence. His cystourethroscopy reveals a bladder neck contracture. Should surgical treatment with an artificial urinary sphincter be offered? Your answer must be in one paragraph and a maximum of 200 words.**
**GPT 4o** **1,0**	No, an artificial urinary sphincter (AUS) should not be immediately offered without first addressing the bladder neck contracture. The presence of a bladder neck contracture can compromise the function of the AUS by affecting urethral dynamics and increasing the risk of obstruction or failure. The contracture should be treated first, typically through endoscopic incision or dilation, followed by a period of observation to ensure resolution and stable voiding. Persistent or recurrent contracture may necessitate additional interventions before considering AUS implantation. Once the bladder neck is adequately treated and the patient demonstrates a stable urethral patency without significant voiding dysfunction, AUS placement can be reconsidered. This stepwise approach optimizes the success of AUS implantation and minimizes complications such as obstruction or erosion.
**DeepSeek** **0,5**	Yes, surgical treatment with an artificial urinary sphincter (AUS) should be offered to this patient. He has severe stress urinary incontinence (SUI) following radical prostatectomy, which has persisted despite conservative treatments, making him a suitable candidate for surgical intervention. The AUS is considered the gold standard for managing post-prostatectomy SUI, particularly in cases of severe incontinence, as it provides effective and durable continence outcomes. The presence of a bladder neck contracture (BNC) does not preclude AUS implantation but must be addressed prior to or during the procedure. BNC can be managed with bladder neck incision or dilation to ensure proper device function and urethral coaptation. Importantly, the patient's lack of urgency or urge incontinence is favorable, as these symptoms can negatively impact AUS outcomes. Preoperative urodynamic studies may be considered to confirm the diagnosis of pure SUI and rule out detrusor overactivity. Shared decision-making is essential, as the patient must understand the risks (e.g., infection, erosion, mechanical failure) and the need for device manipulation. Overall, given his severe SUI and failure of conservative measures, an AUS is a appropriate and effective treatment option.
**Question 19**	**A 70-year-old male patient underwent artificial urinary sphincter implantation five years ago. He remained continent for three years but he has recurrent stress urinary incontinence for the past two years. Proper evaluation confirmed that his incontinence is due to pure stress urinary incontinence. Should surgical revision of the artificial urinary sphincter be offered? Your answer must be in one paragraph and a maximum of 200 words.**
**GPT 4o** **1,0**	Yes, surgical revision of the artificial urinary sphincter (AUS) should be offered to this patient. Given his history of successful continence for three years followed by recurrent stress urinary incontinence, the most likely causes are urethral atrophy or mechanical failure of the device. Proper evaluation, including physical examination, cystourethroscopy, and radiographic assessment, should confirm the integrity of the AUS components and rule out erosion or infection. If the device remains intact, urethral atrophy is the most probable etiology, which may be managed by downsizing the cuff or translocating it to a proximal location. If mechanical failure is identified, complete device replacement is warranted. Since the patient has previously benefited from the AUS and his incontinence is due to stress urinary incontinence alone, revision surgery can restore continence and improve quality of life.
**DeepSeek** **0,5**	Yes, surgical revision of the artificial urinary sphincter (AUS) should be considered for this patient. Recurrent stress urinary incontinence (SUI) after initial success with an AUS is often due to device-related issues such as mechanical failure, urethral atrophy, or cuff erosion. Given that the patient has pure SUI and previously experienced three years of continence, it is likely that the current incontinence is related to a malfunction or degradation of the AUS components. Surgical revision typically involves replacing or repositioning the cuff, pump, or pressure-regulating balloon, depending on the underlying cause. Revision surgery has been shown to restore continence in a significant proportion of patients, with success rates comparable to primary implantation. However, the decision should be individualized, considering the patient's overall health, surgical risk, and preferences. Preoperative evaluation, including urodynamic studies and imaging, is essential to confirm the diagnosis and guide the surgical approach. If the patient is a suitable candidate, revision surgery offers a reasonable chance of restoring continence and improving quality of life.
**Question 20**	**A 58-year-old male patient underwent removal of an artificial urinary sphincter due to urethral erosion six weeks ago. He is bothered with severe stress urinary incontinence and desires reimplantation of the device. Cystourethroscopy does not reveal urethral abnormalities. Should artificial urinary sphincter reimplantation be offered at this moment, considering that the patient is miserable with his incontinence? Your answer must be in one paragraph and a maximum of 200 words.**
**GPT 4o** **1,0**	No, artificial urinary sphincter (AUS) reimplantation should not be performed at this moment. Although the patient is experiencing severe stress urinary incontinence and is eager for reimplantation, a minimum waiting period of 3 to 6 months after AUS explantation due to urethral erosion is generally recommended to allow adequate tissue healing and reduce the risk of recurrent erosion. Early reimplantation may increase the likelihood of complications, including poor tissue integration and repeat erosion. The absence of visible urethral abnormalities on cystourethroscopy is encouraging but does not guarantee complete mucosal and submucosal healing. During the waiting period, the patient should be counseled on temporary management strategies, such as external collection devices or incontinence clamps, to improve quality of life. If severe bother persists, a discussion regarding alternative interventions, such as an adjustable male sling, may be warranted. However, if the patient remains a strong candidate for AUS, reassessment after the appropriate healing period should be performed, including repeat cystourethroscopy, before proceeding with reimplantation.
**DeepSeek** **1,0**	Reimplantation of an artificial urinary sphincter (AUS) should be approached cautiously in this patient, despite his severe stress urinary incontinence and desire for the device. Urethral erosion, which led to the initial removal, indicates compromised urethral integrity, and reimplantation too soon could risk recurrent erosion or infection. Although cystourethroscopy shows no current abnormalities, the urethra may still be healing, and a minimum waiting period of 3–6 months is typically recommended to ensure adequate tissue recovery. Additionally, the patient's history of erosion suggests potential risk factors such as poor tissue quality or compromised vascular supply, which must be carefully evaluated. Alternative management options, such as pelvic floor physiotherapy or a temporary male sling, could be considered to alleviate symptoms while allowing further healing. If reimplantation is pursued, a thorough assessment of urethral health, including urodynamic studies and possibly imaging, is essential to minimize risks. Shared decision-making with the patient, emphasizing the balance between his quality of life and the potential for complications, is crucial. If the urethra is deemed healthy and sufficient time has passed, reimplantation may be appropriate, but premature intervention should be avoided.
